# Clinical Significance of PDCD4 in Melanoma by Subcellular Expression and in Tumor-Associated Immune Cells

**DOI:** 10.3390/cancers13051049

**Published:** 2021-03-02

**Authors:** Thuy T. Tran, Chetan K. Rane, Christopher R. Zito, Sarah A. Weiss, Shlomit Jessel, Liliana Lucca, Benjamin Y. Lu, Victor O. Oria, Adebowale Adeniran, Veronica L. Chiang, Sacit Bulent Omay, David A. Hafler, Harriet M. Kluger, Lucia B. Jilaveanu

**Affiliations:** 1 Department of Medicine (Medical Oncology), Yale University School of Medicine, New Haven, CT 06510, USA; thuy.tran@yale.edu (T.T.T.); chetan.rane@yale.edu (C.K.R.); czito@usj.edu (C.R.Z.); sarah.weiss.sw842@yale.edu (S.A.W.); shlomit.jessel@yale.edu (S.J.); benjamin.lu@yale.edu (B.Y.L.); victor.oria@yale.edu (V.O.O.); harriet.kluger@yale.edu (H.M.K.); 2 Department of Biology, School of Arts, Sciences, Business, and Education, University of Saint Joseph, West Hartford, CT 06117, USA; 3 Department of Neurology, Yale University School of Medicine, New Haven, CT 06510, USA; liliana.lucca@yale.edu (L.L.); david.hafler@yale.edu (D.A.H.); 4 Department of Pathology, Yale University School of Medicine, New Haven, CT 06510, USA; adebowale.adeniran@yale.edu; 5 Department of Neurosurgery, Yale University School of Medicine, New Haven, CT 06510, USA; veronica.chiang@yale.edu (V.L.C.); sacit.omay@yale.edu (S.B.O.)

**Keywords:** PDCD4, brain metastasis, melanoma, PLEKHA5, tumor-infiltrating leukocytes

## Abstract

**Simple Summary:**

While targeting programmed cell death (PDCD) 1 is a central treatment against melanoma, little is known about the related protein PDCD4. We defined differences in melanoma PDCD4 subcellular localization (either total cellular or nuclear-only) during oncogenesis, evaluated its presence on tumor-infiltrating immune cells, and determined its impact on survival. High PDCD4 expression resulted in improved survival in patients with primary and intracranial but not extracranial metastatic melanoma. High PDCD4 levels in surrounding tumor tissue were also associated with increased infiltrating immune cells. PDCD4 may be a potentially useful biomarker in melanoma to help guide our understanding of patient prognosis. Methods to increase PDCD4 in those with melanoma brain metastases may also help improve disease response.

**Abstract:**

Little is known about the subcellular localization and function of programmed cell death 4 (PDCD4) in melanoma. Our past studies suggest PDCD4 interacts with Pleckstrin Homology Domain Containing A5 (PLEKHA5) to influence melanoma brain metastasis outcomes, as high intracranial PDCD4 expression leads to improved survival. We aimed to define the subcellular distribution of PDCD4 in melanoma and in the tumor microenvironment during neoplastic progression and its impact on clinical outcomes. We analyzed multiple tissue microarrays with well-annotated clinicopathological variables using quantitative immunofluorescence and evaluated single-cell RNA-sequencing on a brain metastasis sample to characterize PDCD4+ immune cell subsets. We demonstrate differences in PDCD4 expression during neoplastic progression, with high tumor and stromal PDCD4 levels associated with improved survival in primary melanomas and in intracranial metastases, but not in extracranial metastatic disease. While the expression of PDCD4 is well-documented on CD8+ T cells and natural killer cells, we show that it is also found on B cells and mast cells. PDCD4 expression in the tumor microenvironment is associated with increased immune cell infiltration. Further studies are needed to define the interaction of PDCD4 and PLEKHA5 and to evaluate the utility of this pathway as a therapeutic target in melanoma brain metastasis.

## 1. Introduction

Melanoma is responsible for the majority of skin cancer-related deaths and has a high propensity to metastasize to the brain [1]. In the past decade, systemic and local therapies including targeted therapy, immune checkpoint inhibitors, and stereotactic radiosurgery have significantly improved survival in patients with melanoma brain metastases but are only partially successful due to limited drug penetration through the blood–brain barrier and/or treatment-associated toxicities [2,3,4,5,6,7,8,9,10,11,12]. To date, few molecules have been specifically linked to melanoma cerebrotropism, highlighting this as an area in critical need of further research [13,14,15,16,17,18,19,20,21]. Uncovering key molecules involved in the process of brain metastasis and elucidating pathways associated with them is important given the potential for identifying novel drug targets and developing new therapeutic strategies to counter treatment resistance and enrich management options for this patient population.

We previously established Pleckstrin Homology Domain Containing A5 (*PLEKHA5*), a gene involved in normal brain development, as a regulator of melanoma growth in brain metastasis [22]. Our data suggested that PLEKHA5-mediated growth could be attributed to its role in regulating the cell cycle inhibitor, programmed cell death 4 (PDCD4), via crosstalk with the ubiquitin-proteasome and phosphoinositide 3-kinase/protein kinase B/mammalian target of rapamycin (PI3K/AKT/mTOR) signaling pathways critical in PDCD4 complex regulation [22,23,24,25,26,27,28]. Our study of paired cranial and extracranial human melanomas showed that high PDCD4 expression in cerebral specimens is significantly associated with improved survival, suggesting a role for PDCD4 loss in dysregulating melanoma brain metastasis [22].

PDCD4 was initially identified as a nuclear antigen gene on chromosome 10q24 [29]. PDCD4 is a tumor suppressor downregulated or absent in multiple tumors such as melanoma, lung cancer, hepatocellular carcinoma, breast carcinoma, colorectal cancer, and gastric cancer [23,29,30]. PDCD4 expression is regulated by various pathways, while its biological functions are cell-type-dependent [23,29]. PDCD4 can inhibit neoplastic transformation, tumor angiogenesis and invasion, and/or induce apoptosis [23,29]. Furthermore, PDCD4 downregulation in tumor cells is associated with drug resistance, whereas its expression enhances sensitivity to chemo- and radio-therapy [24,31,32,33,34]. Besides its primary function in carcinogenesis, a new role for PDCD4, as a regulator of the inflammatory process has recently emerged [29,35]. PDCD4 downregulation is associated with an anti-inflammatory phenotype [29,35]. MicroRNA-mediated inhibition of PDCD4 expression enhances the production the anti-inflammatory factor, interleukin (IL)-10, and suppresses expression of pro-inflammatory factors, including IL-6 and tumor necrosis factor-α (TNF-α). Besides tumor cells, PDCD4 is also expressed by cytotoxic T-lymphocytes where its expression is regulated by CTLA-4 and important for T-cell differentiation [36].

Loss or downregulation of PDCD4 is associated with tumor progression and poor outcomes in different malignancies, such as head and neck, brain, breast, lung, digestive, reproductive, and urinary system cancers [23,24,25,26,27,28,37]. In melanoma, the clinicopathological significance and prognostic value of PDCD4 expression have not been thoroughly evaluated. In a small cohort of primary melanomas, low cellular PDCD4 mRNA levels correlated with increased tumor size, high Clark level, and lymph node metastases [30]. Our group reported prognostic significance of total PDCD4 protein levels (cytoplasmic and nuclear) by quantitative immunofluorescence (QIF) in a small cohort of melanoma metastases and shorter survival among patients with absent or low levels of PDCD4 in cerebral lesions [22].

PDCD4 is expressed in the nucleus and cytoplasm of both normal and malignant cells. In tumor cells, PDCD4 shuttles between the two compartments, and its nuclear localization and total protein expression are lost during disease progression [38,39]. We previously found predominantly cytoplasmic PDCD4 staining in cerebral compared to paired extracerebral metastases [22]. However, the sample size of our study (*n* = 37) was not large enough to define the prognostic value of PDCD4 staining patterns in stratified analyses. To our knowledge, prior studies have only evaluated the total PDCD4 protein levels in tissue samples [37]. Only one previous study has evaluated PDCD4 immunohistochemical expression patterns, nuclear vs. cytoplasmic, in pancreatic ductal adenocarcinoma prognosis, but this study was based on a small patient cohort, and PDCD4 subcellular expression levels were measured using semiquantitative methods [37,38]. Given that PDCD4 subcellular localization varies with disease progression, the precise distinction between nuclear and cytoplasmic PDCD4 protein levels at different stages of disease is critical when assessing its clinical significance. Moreover, little is known about PDCD4 expression in the tumor microenvironment. Our study aimed to thoroughly characterize PDCD4 expression in human melanoma specimens and benign nevi and to evaluate the prognostic role of PDCD4 in tumor vs. immune-infiltrating cells using a large historical cohort of primary and metastatic melanoma specimens annotated for clinical outcome.

## 2. Materials and Methods

### 2.1. Tissue Microarray (TMA) Construction

Melanoma and nevi TMAs were constructed from a historical cohort, as described previously [40,41,42]. In brief, paraffin-embedded, formalin-fixed (FFPE) tissue blocks were obtained from the Yale University Department of Pathology Archives with clinical data as approved by the Yale University Institutional Review Board. Representative regions of invasive tumor were identified by a pathologist, and 0.6 mm diameter cores were obtained from each specimen and integrated into a TMA.

The large melanoma array included 230 primary and 293 metastatic melanomas resected between 1959 and 2000, 55% were from male patients, mean follow-up of 6.7 years (range 2 months to 40 years), and mean age at diagnosis was 52.4 years (range 18–91 years). The nevus array contained cores from 263 benign lesions as well as 40 metastatic or primary specimens from patients that were also represented on the aforementioned melanoma array. Both arrays contained identical cell lines, cored from cell pellets, as previously described [41,42]. The overlapping metastatic and primary specimens and cell lines were used for normalization of the scores obtained from the benign and malignant arrays.

The matched cerebral-extracranial melanoma array consisted of samples from 37 patients who underwent craniotomy between 1997 and 2014. Patient tumor characteristics and clinical information for the 37 patients have been previously described [40]. Briefly, mean age at diagnosis was 51 years (range 19–78 years) and included 68% males. Survival time was calculated as the time from first distant metastasis diagnosis to death or last follow-up (mean 1.8 years; follow-up range 0.22–9.8 years) and from first melanoma brain metastasis diagnosis to death or last follow-up (mean 1.2 years; follow-up range 0.13—9.8 years). Brain metastasis-free survival was defined as time of stage IV diagnosis until brain metastasis diagnosis (mean 0.52 years; follow-up range 0–3.6 years).

### 2.2. Immunofluorescent Detection of PDCD4

Five µm TMA sections were mounted on glass slides using an adhesive tape-transfer system with ultraviolet cross-linking and subjected to immunohistochemical staining. Briefly, TMA slides were deparaffinized, hydrated in graded alcohol washes, and subjected to Tris-ethylenediaminetetraacetic acid (pH 8.0, Santa Cruz Biotechnology, Dallas, TX, USA, sc-296654) antigen retrieval by boiling for 20 min in a pressure cooker. Endogenous peroxidase activity was blocked using hydrogen peroxide solution. Unspecific staining was blocked in 0.3% bovine serum albumin solution before slides were concomitantly stained with a cocktail of rabbit anti-PDCD4 antibody (Cell Signaling, Danvers, MA, USA, D29C6) and either mouse anti-S100 (BioGenex, San Ramon, CA, USA, clone 15E2E2, MU058-UC) and mouse anti-HMB45 (BioGenex, MU001A-UC) to detect tumor cells or mouse anti-CD3 (Leica Biosystems, Wetzlar, Germany, CD3-565-L-CE) to detect tumor-infiltrating lymphocytes. Separate serial slide cuts were stained with mouse anti-CD8 (Dako, Glostrup, Denmark, M7103), rabbit anti-CD4 (SpringBio, Pleasanton, CA, USA, M3352), and mouse anti-FOXP3 (Abcam, Cambridge, MA, USA, ab20034) for T cell subsets, mouse anti-CD20 (Dako, M0755) for B-cell detection, and mouse anti-CD68 (Dako, M0814) to detect macrophages. Goat anti-rabbit or anti-mouse amplification reagent (Envision; Dako, K4003) was utilized to amplify the signal, visualized with Cyanine-3- or Cyanine-5-tyramide (Perkin Elmer, Waltham, MA, USA, SAT705A001EA). Goat anti-mouse IgG conjugated to Alexa 546 (ThermoFisher Scientific, Waltham, MA, USA, A-11030) was used for visualization of S100 and HMB45 staining. Nuclei were identified by 4,6-diamidine-2-phenylindole (DAPI) staining. Coverslips were mounted with ProLong Gold antifade medium (Invitrogen/Life Technologies, Carlsbad, CA, USA, P36931).

### 2.3. Quantitative Determination of PDCD4 Expression

Image capturing and quantitative measurements were conducted using methods previously described [40,43,44]. Tumor was distinguished from the surrounding stromal elements via automated processing and thresholding of the S100/HMB45 signal to generate a tumor mask. A total tissue mask (tumor and stroma) was defined using the DAPI+ nuclear compartment. The stromal compartment was obtained by subtracting the tumor mask from the total tissue mask. The CD3 image was used to define the total T cell area. For quantification of PDCD4 expression, signal in the tumor or stromal compartments or CD3-positive T cell area was expressed as average intensity on a scale of 0–255. To assess the degree of tumor-infiltrating lymphocytes density, we used the percentage of either CD3-, CD4-, CD8-, FOXP3-, or CD20-positive area within the histospot. For macrophages, we used the CD68-positive area. Tumor spots were excluded if they contained insufficient tissue (<3% of the histospot area) or abundant necrotic tissue.

### 2.4. Statistical Analysis

JMP version 5.0 software was used (SAS Institute, Cary, NC, USA). Data was analyzed using either continuous immunofluorescence scores or variables dichotomized at the median. The two-sample t test (analysis of variance) for continuous measurements and the Chi Square test for dichotomized variables were used to test relationships between PDCD4 expression levels or sub-cellular localization and tumor type, site of metastasis (intracerebral and extracerebral), or intra-tumoral immune cell densities. The prognostic significance was assessed using the Cox proportional hazards model with survival as an end point. Survival curves were generated using the Kaplan–Meier method. The association between continuous QIF measurements and other clinical/pathological parameters was assessed by the two-sample t test (analysis of variance (ANOVA)).

### 2.5. Single-Cell Melanoma Brain Metastasis Sequencing

A melanoma brain metastasis sample was freshly collected as part of standard of care. The patient provided signed informed consent per tissue collection protocol approved by the Yale institutional review board. Tissue was minced and dissociated using Hank’s Balanced Salt Solution medium containing Collagenase IV (2.5 mg/mL) and DNAse I (0.2 mg/mL) (Worthington Biochemical Corporation, Lakewood, NJ, USA) for 30 min at 37 °C, followed by Lymphoprep gradient centrifugation. Cells were stained and sorted for CD45 expression (eBioscience, San Diego, CA, USA, PerCP-Cy5.5 conjugated) and TcRab (eBioscience, PE-Cy7 conjugated) expression on a Becton Dickinson Fluorescence-Activated Cell Sorting Aria II using a live cell (Live/Dead Cell Viability Assay, Life Technologies). 50% Live/CD45+/TcRab+ and 50% Live/CD45+ were mixed together. The single-cell RNA library was prepared and sequenced at the Yale Center for Genome Analysis using established protocols from 10× genomics (https://medicine.yale.edu/keck/ycga/sequencing/10x/singcellsequencing/, accessed on 17 February 2020). scRNAseq libraries were sequenced on an Illumina NovaSeq S4 instrument at a read length of 26 × 8 × 91 base pairs and at a depth of 300 million reads per sample. Data were analyzed using Seurat version 3.0. Cells expressing mitochondrial reads < 15% were retained for analysis, log-normalized, and variable features were identified using 2000 variable genes. Data was scaled using the ScaleData function and principal component analysis was performed to identify 15 components. Uniform ManifoldApproximation and Projection (UMAP) dimensionality reduction and cluster identification were performed with the FindNeighbors function and the FindClusters function (resolution = 0.5). Clusters were annotated based on expression of lineage-defining markers (CD3E, CD4, and CD8A for T cells, MS4A1 for B cells, FOXP3 for Tregs, KIT for mast cells, IGHG3 for plasma cells, C1QA for microglia, CD14 for macrophages, GNLY for NK cells). Genes correlated with PDCD4 expression were identified by calculating the Spearman’s coefficient between the average expression of PDCD4 and the average expression of every gene in the dataset, and by adjusting *p*-values by multiplying them by the number of genes in the dataset.

## 3. Results

### 3.1. Transition from Nuclear to Cytoplasmic PDCD4 Expression Is Linked with Melanoma Development and Progression

PDCD4 is well-documented to have functional significance depending on its expression as either a nuclear or cytoplasmic protein in multiple cancer types [27,45,46]. Although expression changes have been associated with differences in melanoma aggressiveness in vitro, not much is known about the compartmentalization of PDCD4 expression in human melanoma tissue during metastatic progression. We previously noted two distinct PDCD4 staining patterns in metastatic melanoma tissues: cytoplasmic and nuclear or nuclear only. Based on this observation, our current studies evaluated PDCD4 expression either overall in the S100-positive melanocyte/melanoma (simultaneous measurement of expression in the cytoplasm and nuclei—‘total PDCD4′) or the DAPI-positive nuclei (‘nuclear PDCD4′).

**Figure 1:**
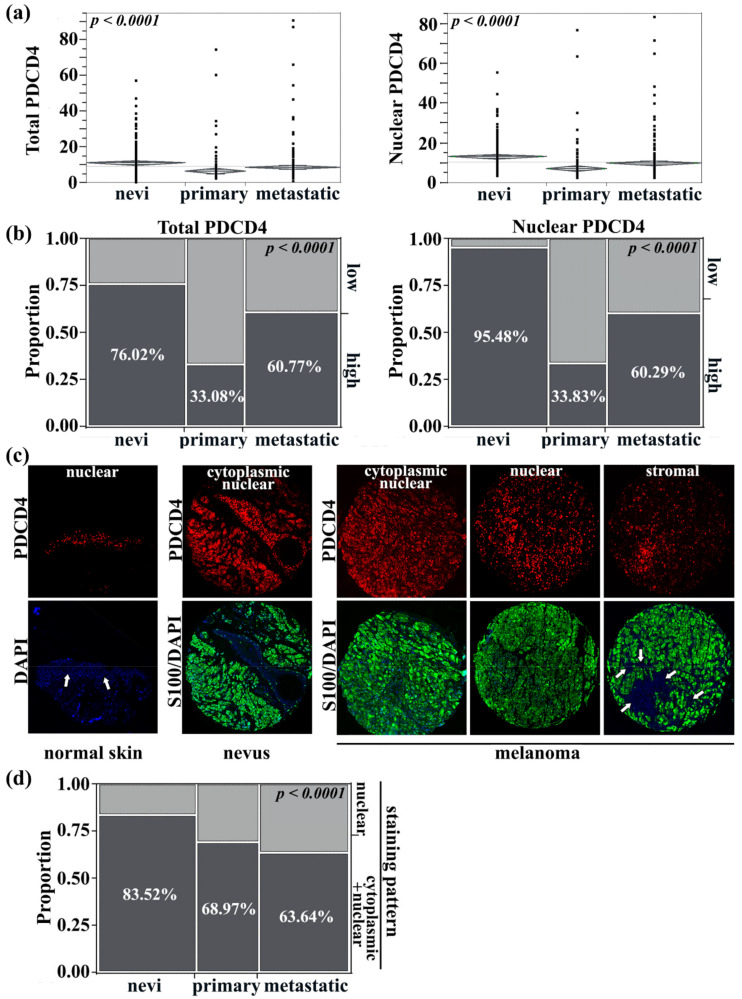
Expression patterns of PDCD4 during melanoma progression. (**a**) PDCD4 expression was higher in both the S100-positive melanocyte/melanoma (‘total PDCD4′) and DAPI-positive nuclear (‘nuclear PDCD4′) compartments of benign tissue compared to malignant tissue (ANOVA, *p* < 0.0001). (**b**) High PDCD4 expression was predominantly in nevi, decreased in primary melanomas, but increased again in metastatic melanomas—this pattern was evident when analyzing total PDCD4 or nuclear PDCD4. (**c**) Representative photos of PDCD4 (red) compartmentalization in the cytoplasmic (green, defined by S100) vs. nuclear (blue) in normal skin vs nevi vs melanoma. For melanoma, three different staining patterns are being shown for PDCD4: cytoplasmic and nuclear, predominantly nuclear, or stromal. White arrows indicate the skin epidermis (left-most panel) or tumor stroma (right-most panel). Magnification 10×. Higher 40× magnification photos of the top row are shown in Figure S1. (**d**) During progression from nevi to primary and then to metastatic melanoma, cytoplasmic/nuclear PDCD4 expression decreases (dark gray) compared to nuclear-only expression (light gray).

Using a large historical cohort of 523 melanoma cases (230 primaries and 293 metastases largely from extracranial sites) and a cohort of 263 benign lesions, we found that PDCD4 expression was significantly higher in benign vs. malignant tissue regardless of subcellular localization (ANOVA, *p* < 0.0001), Figure 1a. When total PDCD4 levels were dichotomized by the median immunofluorescence score into high vs. low staining intensity groups, 168/221 (76.02%) nevi had high PDCD4 expression compared to 44/133 (33.08%) of primary lesions and 127/209 (60.77%) of metastatic melanoma tumors (Chi-square, *p* < 0.0001). When analyzing only the nuclear compartment in these samples, a similar pattern emerged: 95.48% of nevi were high-expressing cases, compared to only 33.83% of primaries and 60.29% of cases in metastatic melanoma tumors (Chi-square, *p* < 0.0001), Figure 1b. These data indicate that PDCD4 expression is altered during neoplastic progression, with higher expression during metastasis. PDCD4 was restricted to the nuclear compartment in normal skin (12/12 cases (100%)), mostly cytoplasmic in nevi, and found in either one or both compartments in melanoma by visual assessment, as shown in Figure 1c, with higher magnification photos in Figure S1. 152/182 (83.52%) of nevi had cytoplasmic/nuclear (‘total’) staining, which then decreases during progression from primary (80/116 of cases (68.97%)) to metastatic melanoma (105/165 of cases (63.64%)), (Chi-square, *p* < 0.0001), Figure 1d. When stratifying PDCD4 expression based on predominant subcellular compartmentalization, we again found significantly higher PDCD4 expression levels in metastatic compared to primary lesions regardless of subcellular localization (Chi-square, *p* < 0.0001 and *p* = 0.0002 respectively, Supplementary Figure S2a,b). There were no 5-year survival differences in primary or metastatic melanomas when stratified by site of PDCD4 subcellular expression (log-rank test, *p* = 0.47 in primary and *p* = 0.39 in metastatic melanomas), Figure S2c,d.

We further evaluated the association between PDCD4 expression pattern and several other clinicopathological variables. As our cohort included cases from 1959 to 2000, these patients were not exposed to modern monoclonal checkpoint inhibitors. Instead, immune-targeted treatments included interferon and high-dose IL-2. Only 11/199 patients with primary tumor for analysis and 48/280 patients with metastatic melanoma received these early versions of immune therapy. When we evaluated if treatment affected PDCD4 subcellular localization, we found that of the patients who received these earlier versions of immunotherapy, more had PDCD4 confined to the nucleus than the cytoplasm (Chi-square *p* = 0.033). The impact of immunotherapy on PDCD4 subcellular localization is however limited by the small number of cases available for this analysis (19 of 48). There was no significant association between PDCD4 expression pattern and other clinicopathological variables.

### 3.2. Higher Levels of PDCD4 Are Associated with Improved 5-Year Survival in Primary but Not in Metastatic Melanomas

**Figure 2:**
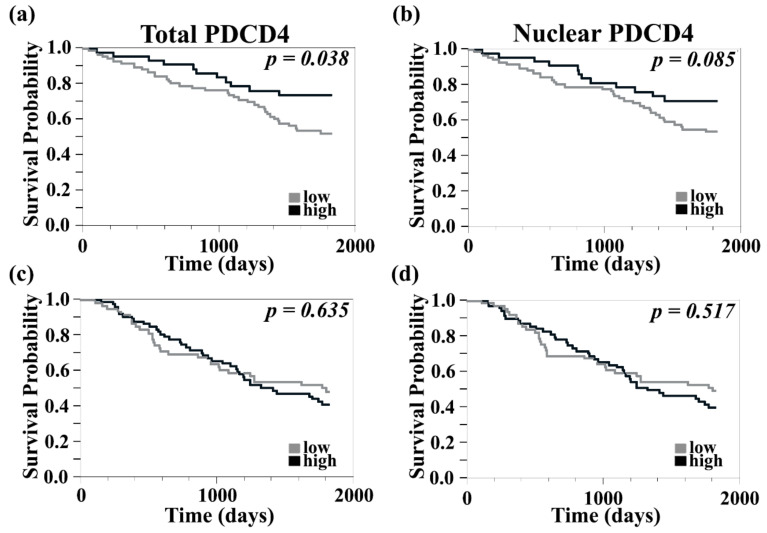
Improved 5-year survival in primary but not metastatic melanomas with high PDCD4 expression. (**a**) The 5-year survival in primary melanomas was improved with high total PDCD4 expression but (**b**) only associated with a trend towards improved survival with high nuclear PDCD4 expression. High PDCD4 expression was not associated with improvements in 5-year survival in metastatic melanoma regardless of whether expression was analyzed within the total tumor (**c**) or the nuclear compartment (**d**).

We previously found that while PDCD4 levels in intracranial metastases were not significantly different when compared to their paired extracranial counterparts, expression patterns differed between sites. PDCD4 was predominantly cytoplasmic in brain lesions. Furthermore, high PDCD4 expression in cerebral specimens, regardless of its subcellular localization, was associated with better survival [22]. We therefore sought to further evaluate the prognostic role of PDCD4 by studying its expression levels in our large primary and metastatic melanoma TMA with relationship to clinicopathological data. PDCD4 QIF scores were dichotomized by the median value into ‘high’ and ‘low’ groups and Kaplan–Meier survival curves were generated. High total PDCD4 expression levels correlated with improved 5-year survival in primary melanomas, Figure 2a (log-rank test, *p* = 0.038; Risk Ratio (RR) 0.704; Lower Confidence Limit (CL) 0.491; Upper CL 0.97, *p* = 0.031). There was a trend towards improved 5-year survival in primary melanomas with high nuclear PDCD4, Figure 2b (log-rank test *p* = 0.085; RR 0.914; Lower CL 0.803; Upper CL 0.998, *p* = 0.043).

Contrary to its association with improved 5-year survival in primary melanomas, PDCD4 expression was not associated with survival differences in metastatic disease either within the total tumor or the nuclear compartment, Figure 2c, d (log-rank *p* = 0.63 and *p* = 0.52, respectively). This result supports our previous findings from a smaller cohort of matched intracranial and extracranial sites, which showed no association between PDCD4 expression and survival in extracranial metastases.

### 3.3. Higher PDCD4 Staining Was Associated with Clark Level and Absence of Microscopic Satellites but Not with Other Features of Primary or Metastatic Melanoma

Given that high PDCD4 levels are associated with improved 5-year survival in primary melanomas, we evaluated additional tissue variables for prognostic significance using *t*-tests. High total and nuclear PDCD4 expression was significantly associated with higher Clark level (non-paired *t*-test, *p* = 0.010 and *p* = 0.011, respectively) and absence of microscopic satellites in primary tumors (non-paired *t*-test, *p* = 0.0093 and *p* = 0.010, respectively). There was no significant association between PDCD4 expression and Breslow primary tumor thickness, presence of ulceration, age, gender, or prior immune-targeted treatment.

### 3.4. PDCD4 in the Tumor Stroma Is Associated with Improved Survival in Primary Melanomas, Correlated with Higher Tumor-Infiltrating Lymphocyte Content, and Identified on CD8+ T Cells and CD20+ B Cells

**Figure 3:**
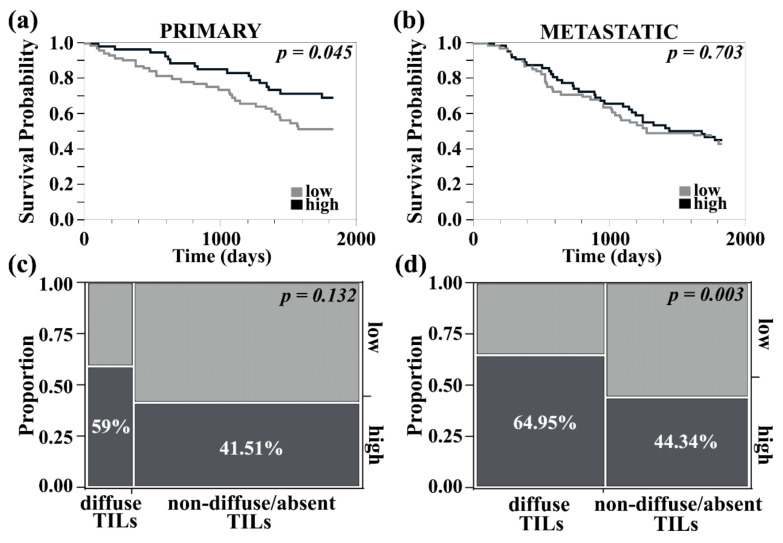
PDCD4 expression in the tumor stroma of primary and metastatic melanoma. (**a**) High PDCD4 expression in primary tumor stroma had improved 5-year survival. (**b**) High PDCD4 expression in metastatic tumor stroma did not impact 5-year survival. (**c**) Stromal PDCD4 was dichotomized into high/low groups and compared with stromal tumor-infiltrating leukocyte quantity (either brisk/diffuse vs. non-diffuse/absent). A trend towards significance was seen between high stromal PDCD4 and brisk/diffuse TILs in primary tumors (Chi-square, *p* = 0.13). (**d**) There were significantly more cases of high stromal PDCD4 that had diffuse TILs in metastatic melanoma (Chi-square, *p* = 0.0031).

As previously reported, staining for PDCD4 was cytoplasmic and/or nuclear in melanoma cells as well as on immune-infiltrating cells in the stroma. We next sought to study stromal PDCD4 expression in primary and metastatic melanoma, its correlation with total and nuclear tumor PDCD4 levels, and its association with survival. Melanoma metastases had increased stromal PDCD4 compared to primary lesions (non-paired *t*-test, *p* = 0.029). There was a strong correlation between stroma and tumor compartment PDCD4 expression in both primary (R^2^ = 0.60, *p* < 0.0001) and metastatic melanomas (R^2^ = 0.73, *p* < 0.0001). Improved 5-year survival was seen in primary melanomas with high stromal PDCD4 expression (log-rank test, *p* = 0.045, RR 0.738; Lower CL 0.54; Upper CL 0.98, *p* = 0.042), Figure 3a. Conversely, metastatic melanoma with high PDCD4 stromal expression was not associated with improved survival (log-rank test, *p* = 0.70), Figure 3b. Stromal PDCD4 levels were not associated with Breslow primary tumor thickness, Clark level, presence of ulceration, age, gender, or prior immune-targeted treatment. However, higher PDCD4 levels were detected in tumors without microsatellites (non-paired *t*-test, *p* = 0.025). In primary melanomas, more cases with higher PDCD4 stromal expression had brisk/diffuse tumor-infiltrating lymphocytes, though the analysis only trended toward significance (Chi-square, *p* = 0.132), Figure 3c. Higher PDCD4 stromal expression was found in metastatic tumors with increased tumor-infiltrating lymphocytes (Chi-square, *p* = 0.003), Figure 3d. PDCD4 is thus associated with T cell infiltration, particularly in metastatic melanoma.

**Figure 4:**
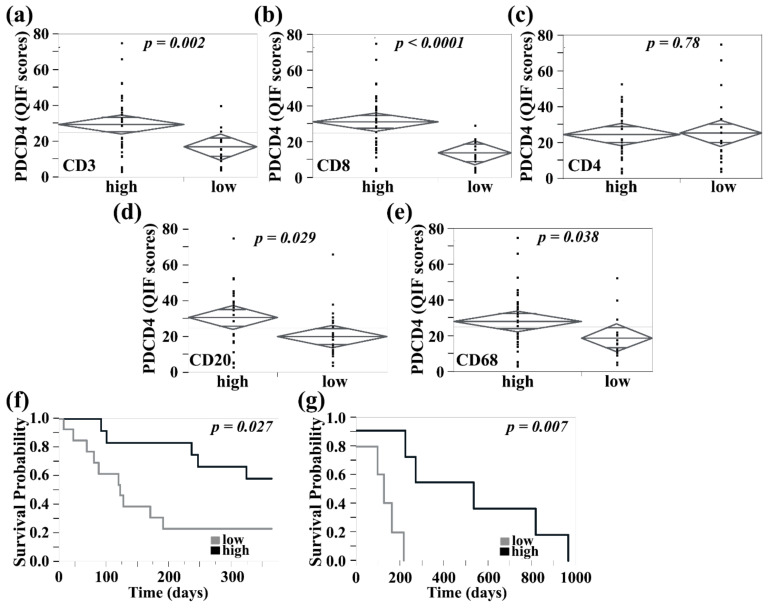
Analysis of PDCD4 expression in tumor infiltrating immune cells. (**a**–**c**) The CD3-, CD8-, and CD4-positive populations were dichotomized into two groups with high and low T cell content. (**a**,**b**) Cases with high CD3 and CD8 were independently found to have high stromal PDCD4 levels (non-paired *t*-test, *p* = 0.002 and *p* < 0.0001, respectively). (**c**) CD4 was not associated with stromal PDCD4 levels. (**d**) The CD20-positive population was dichotomized by the median into two groups with high and low B cell content. Cases with high CD20 B cell density had higher stromal PDCD4 expression (non-paired *t*-test, *p* = 0.029). (**e**) CD68-positive macrophage populations were also dichotomized into two groups with high and low macrophage content. PDCD4 expression was increased in the group with high CD68+ macrophage density (non-paired *t*-test, *p* = 0.038). (**f**) High PDCD4 expression within the total tumor compartment was associated with improved 1-year survival from the date of brain metastasis diagnosis (log-rank test, *p* = 0.024). (**g**) Evaluation of PDCD4 expression in intracranial CD3+ T cells shows that high PDCD4 was associated with improved brain metastasis-free survival, defined as the time from the date of stage IV diagnosis to brain metastasis diagnosis (log-rank test, *p* = 0.007).

Using a TMA containing 37 paired extracranial and intracranial metastases, PDCD4 was co-stained with CD3 to allow quantification of PDCD4 within the CD3-positive compartment. We previously showed that PDCD4 levels in cerebral metastases are not significantly different when compared to their extra-cerebral counterparts. Consistent with our previous findings, there was no difference in PDCD4 expression within the total tissue compartment (defined by DAPI signal and including stroma and tumor) or CD3-positive compartment, between brain lesions and extracranial sites (paired *t*-test, *p* = 0.35 and *p* = 0.90, respectively). We next evaluated the association between PDCD4 expression and density of tumor-infiltrating lymphocytes as measured by CD3 (for T cells) or CD20 (for B cells) positivity, and macrophages as measured by CD68 positivity. Tumor-infiltrating lymphocytes and macrophage populations were split in two groups with high and low content determined by the median % area. Two-sample *t*-test of continuous PDCD4 intensity scores showed that high stromal PDCD4 expression was associated with high CD3+ and CD8+ T cell subsets, with CD20+ B cells, and with CD68+ macrophage densities, within intracerebral and extracerebral metastases (non-paired *t*-test, *p* = 0.002, *p* < 0.0001, *p* = 0.029, *p* = 0.038, respectively). The association was not statistically significant for CD4+ T cells regardless of metastatic location (Figure 4a–e).

We next evaluated PDCD4 expression in association with survival in the tumor and stroma of brain metastases. Individuals with high total tissue PDCD4 expression had longer survival from the time of brain metastasis diagnosis (log-rank test, *p* = 0.026; RR 0.54; Lower CL 0.3; Upper CL 0.93) (Figure 4f), and the same was seen in cases with high nuclear PDCD4 tumor cell expression (log-rank test, *p* = 0.004). When evaluating PDCD4 expression, specifically in intracranial CD3+ T cells, high PDCD4 expression was associated with further improvement in brain metastasis-free survival (log-rank test, *p* = 0.007; RR 0.3; Lower CL 0.068; Upper CL 0.75), Figure 4g. To assess the independent predictive value of PDCD4 expression in tumor and stroma, we performed Cox proportional hazards analysis, which included as covariates treatment information and BRAF/NRAS mutational status. Our cohort consisted of cases collected between 1997 and 2014, and treatment information was available for 31 cases. Of these, 14 patients received newer therapies, which mostly consisted of checkpoint inhibitors, and 13 cases had either BRAF or NRAS mutation. PDCD4 expression in both tumor and nearby CD3+ T cells did not associate with either mutational status or administration of modern treatment regimens and remained an independent prognostic indicator. Although these are interesting findings, both the impacts of mutational status and immunotherapy are limited by the small number of patients represented.

### 3.5. PDCD4 Expression in Immune Cells Reveal Expression on CD8+ T, B, NK, and Mast Cells

**Figure 5:**
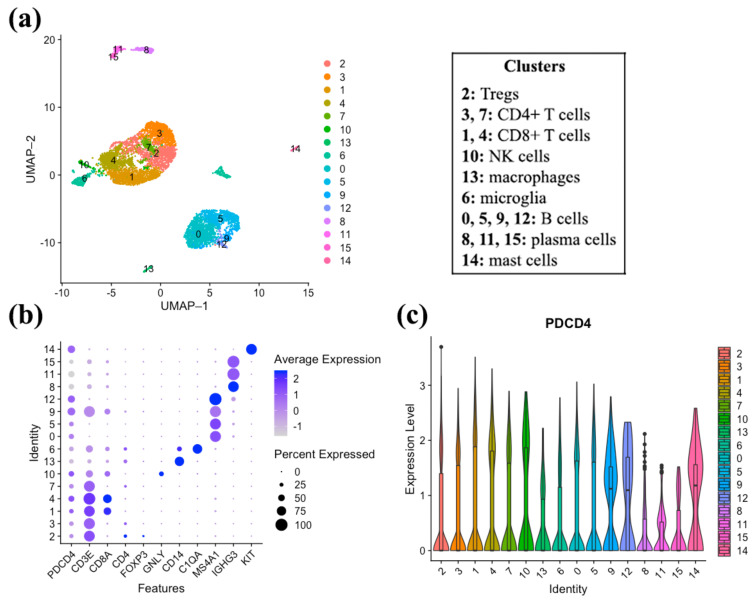
Single-cell sequencing reveals PDCD4 expression on cytotoxic immune cells. (**a**) Uniform ManifoldApproximation and Projection (UMAP) image displaying clustered cell populations. (**b**) PDCD4 expression is highest in CD8+ T cells (clusters 1 and 4 based on CD3E and CD8A expression), B cells (clusters 0, 5, and 12 based on MS4A1 expression), NK cells (cluster 10 based on GNLY expression), and mast cells (cluster 14 based on KIT expression). Of note, cluster 9 expressed genes which overlap with CD8+ T cells and B cells based on expression of CD3E, CD8A, and MS4A1. (**c**) PDCD4 expression level by cell cluster.

To further characterize the immune subtypes in melanoma brain metastasis that express PDCD4, we used 10X transcriptomic single-cell sequencing to identify key immune populations. Using CD45+-sorted immune cells in the brain of a resected melanoma brain metastasis, we found that PDCD4 is expressed on CD8+ T cells and NK cells as anticipated, but also demonstrate novel expression on B cells and mast cells (Figure 5a,b). In T cells, PDCD4 expression was highly correlated with features of cytotoxicity (high granzyme levels (GZMK and GZMA, *p* < 0.001), NKG7 (*p* = 0.0023), and CTSW (*p* = 0.028)), Table S1. In B cells, PDCD4 expression was also associated with cytotoxicity (GZMA *p* = 0.011), Table S2.

## 4. Discussion

The role of PDCD4 in cancer progression is still being defined. Only a handful of studies have evaluated PDCD4 expression in human melanomas, primarily using cell lines absent of clinical data [47]. We offer insight into the subcellular distribution of PDCD4 in melanoma progression and delineated differences between extracranial and intracranial metastases. Our study is unique in multiple aspects, as no prior studies have (1) examined PDCD4 levels in clinical specimens using an automated method of expression analysis, (2) distinguished differences in PDCD4 subcellular localization and related localization to clinical outcomes, or (3) characterized its expression between tumor vs. immune cells. Our method provides precise, reproducible measurement of antigen levels, free of the subjectivity associated with pathologist-based scoring employed in traditional immunohistochemistry studies.

PDCD4 binds RNA and is suspected to have a role in RNA metabolism and protein translation. It is thought to be actively exported from the nucleus via a Crm-1-dependent mechanism in response to cellular stress [45]. A study showed decreased PDCD4 mRNA levels in melanoma compared to adjacent normal tissue but did not differentiate between nuclear vs. cytoplasmic PDCD4 compartmentalization. It also suggested that in vitro overexpression led to decreased proliferation, increased apoptosis, and decreased melanoma cell invasion [48]. These findings are congruent with our prior results, indicating an inverse relationship between PDCD4 protein levels and proliferation in patient melanomas. MicroRNAs (miR), such as miRNA-21, are known to transcriptionally suppress several tumor suppressor genes, such as PTEN and PDCD4, and increased miRNA-21 augments metastatic burden in a B16 mouse melanoma model by promoting cancer cell proliferation, survival, and metastasis [49]. Furthermore, PDCD4 is translationally and post-translationally regulated, so mRNA is less accurate than protein levels in determining its pathologic impact. This may explain why the The Cancer Genome Atlas skin melanoma fails to show a correlation between PDCD4 and PLEKHA5 mRNA levels. In our previous study of cerebrotropic melanomas, we found the mechanism accounting for PDCD4 loss or suppression was post-translational, via proteasomal degradation and likely regulated by the crosstalk of PLEKHA5, a mediator of brain metastasis, with the PI3K/AKT and ubiquitin-proteasome pathways [22].

The exact mechanism of PDCD4 regulation and interaction with PLEKHA5 is yet to be elucidated and is the focus of ongoing studies. PDCD4 intracellular trafficking is regulated in a context-dependent manner by both AKT/P70S6K or RAS/MAPK pathway signaling [50,51,52]. Interestingly Mudduluru et al. found an inverse correlation between nuclear PDCD4 levels and nuclear phosphorylated-AKT (pAKT) levels in colon carcinoma samples [53]. Moreover, the transition from nuclear to cytoplasmic PDCD4 staining was significantly associated with high pAKT staining in the nucleus and cytoplasm. Increased PI3K/AKT pathway activation was found in melanoma brain metastases when compared to matched extracranial sites [54,55]. In contrast, MAPK pathway signaling was not altered between these paired sites. Hyperactivation of PI3K/AKT pathway in response to stimuli from the intracranial microenvironment might be responsible for PDCD4 translocation from the nucleus to the cytoplasm and studies focused on PI3K/AKT signaling dynamics in relation to PLEKHA5 expression and PDCD4 intracellular localization in cerebral tumors compared to extracranial counterparts are warranted.

Although overall PDCD4 expression decreases during the transition from nevi to melanoma, we believe the shuttling from nuclear to cytoplasmic subcellular localization is more pathologically relevant in disease progression. Here, we found that PDCD4 is restricted to the nuclear compartment in normal skin but transitions mainly to cytoplasmic expression in nevi, primary melanomas, and metastatic melanomas, suggesting a role in melanoma disease progression. We previously reported that PDCD4 staining was predominately cytoplasmic in cerebral compared to extracerebral melanoma metastases. We present findings using a larger cohort of cases that is consistent with previous studies showing that PDCD4 translocation from the nucleus to the cytoplasm is associated with disease progression [27,56]. A similar switch in localization was associated with tumor progression in colon adenocarcinoma [57], thus suggesting cytoplasmic PDCD4 is a hallmark of progressive cancer across multiple malignancies. When PDCD4 QIF scores without regard to subcellular localization were evaluated between brain and extracranial metastases, no differences were seen. However, differences emerged following further characterization of PDCD4 subcellular localization, thus emphasizing the importance of making this distinction. The intensity of staining, a reflection of protein quantity, increased slightly with progression from primary to metastatic melanoma regardless of its subcellular localization. Additionally, higher PDCD4 expression was seen with higher Clark level and decreased microscopic satellites in primary melanomas. It is unclear though how PDCD4 contributes to Clark level, particularly as Clark level is not considered during staging, but it is possible that PDCD4 upregulation may be employed to control local tumor aggressiveness. Elevated PDCD4 levels in cases with absent microsatellite disease are consistent with this notion. The highest PDCD4 expression was detected in nevi, which supports its potential role as an inhibitor of malignant transformation.

Our previously reported data on PLEKHA5 as a regulator of metastatic melanoma growth indicated an inverse relationship between PLEKHA5 and PDCD4. Additionally, while PLEKHA5 was positively correlated with Ki-67 staining, PDCD4 had a negative correlation with proliferation. We have now found that 5-year survival was improved in primary melanoma cases with high PDCD4 expression, but no survival differences were seen when evaluating PDCD4 expression in metastatic disease using a TMA primarily consisting of extracranial metastases. We showed that higher PDCD4 levels are associated with improved metastasis-free survival in patients and can serve as a biomarker of disease. Further studies on the use of PDCD4 as a prognostic biomarker are warranted. There may also be a role for PDCD4 as a predictive biomarker given its potential impact on sensitizing tumors to anti-neoplastic agents and radiation [24,31,32,33,34,58].

We further characterized PDCD4 expression on immune cells in the tumor stroma, as little is known in this area. PDCD4 has previously been documented to be present on CD4+ [59] and CD8+ T cells and NK cells [60] in other disease models. Loss of PDCD4 in a murine model of hyperlipidemia was associated with decreased CD8+ T cells and increased regulatory T cells [61]. Based on single-cell sequencing data on a melanoma brain metastasis sample, we found that PDCD4 was also present on B cells and mast cells, which has never been previously reported. Although mast cells are rare in the normal brain, our enrichment for CD45+ immune cells enabled us to capture this rare population, which may be elevated in pathological states. We also found that PDCD4 expression is higher in CD8+ compared to CD4+ T cells. PDCD4 expression in these cells was positively correlated with genes involved in cytolysis, thus potentially supporting the role of PDCD4 in immune-directed cell lysis of tumor cells and explaining how high PDCD4 levels are associated with improved brain metastasis survival. Although we found an association between CD68+ macrophages and high PDCD4 expression using QIF, PDCD4 was not present at high levels in the macrophage (defined as CD14+/C1QA−) or microglia (defined as C1QA+) populations in our one sequenced melanoma brain metastasis sample. We found that PDCD4 is increased in the stroma of metastatic compared to primary melanoma, but expression did not translate into differences in survival. However, increased stromal PDCD4 expression in primary melanomas was associated with an improvement in 5-year survival. In intracranial metastases, high PDCD4 expression within the total tissue compartment led to improved survival from the date of brain metastasis diagnosis. As we know that PDCD4 is expressed in cytotoxic CD8+ T cells, this may explain the improved survival in primary melanomas. However, further studies are needed to identify the specific immune subset responsible for driving these clinical outcomes. Additionally, the expression and role of PDCD4 on B cells and mast cells will need to be elucidated in the context of anti-tumor immunity. Furthermore, miR-21 has been shown to act as a negative modulator of T cell activation with suppression of memory function [62]. In stimulated naïve CD4+ T cells, miR-21 loss was found to increase PLEKHA1 [62]. Given the relationship between PLEKHA1 and PLEKHA5, it is reasonable to investigate whether miR-21 also regulates PLEKHA5 expression and confirm its relationship to PDCD4 in inflammatory cells.

Our studies used tissue collected before the widespread use of modern checkpoint inhibitors. Many individuals received earlier immuno-therapeutics like interferon and high-dose IL-2. The utility of PDCD4 as a biomarker for melanoma response to current checkpoint inhibitors will need to be further validated as a result. Additionally, the large melanoma array did not contain matched primary and metastatic melanomas from the same patients as they progressed in their disease course. It is unclear how PDCD4 expression changes with treatment.

PDCD4 has complex roles dependent on not only its subcellular site of expression but also on whether it is expressed on tumor or immune cells in the microenvironment. Future studies will focus on the interaction of PLEKHA5 and PDCD4 and the critical role of PDCD4 on boosting anti-tumor responses and survival in brain metastases.

## 5. Conclusions

We showed that PDCD4 may be involved early on during the critical process of neoplastic transformation. PDCD4 is most highly expressed in nevi but levels increase again during metastasis with a concurrent shift from nuclear to cytoplasmic localization of the protein. High tumor and stromal PDCD4 levels are associated with improved survival in primary melanomas and in intracranial metastases, but not in extracranial metastatic disease. Our data suggested that the survival benefit of PDCD4 in intracranial metastases is present regardless of where PDCD4 is expressed in the tumor cells (either nuclear or in the nucleus/cytoplasm) or stroma. We also confirmed that PDCD4 is expressed by key cytolytic cells in the melanoma brain tumor microenvironment, namely CD8+ T cells and NK cells. We further showed that significant levels are also found on B cells that harbor cytolytic activity and mast cells in brain metastases, which was previously unknown. PDCD4 expression is strongly correlated between tumor and stroma of primary and metastatic melanomas, where it is associated with increased immune infiltration. Finally, higher PDCD4 expression in CD3+ T cells translated into improved brain metastasis-free survival. It will be critical to understand how PDCD4 can be employed as a prognostic biomarker in intracranial disease and how it can be therapeutically targeted to improve survival in melanoma patients.

## Data Availability

Single-cell sequencing data was shared with us for our manuscript but collectively owned by a number of investigators at Yale focused on studying immune populations in brain metastases. This dataset belongs to a larger series for which the group has not finished analysis, and thus restrictions apply to the availability of the data. Upon completion of analysis, they will be collectively and publicly deposited. The data may be available from the authors upon reasonable request and with permission of the Yale brain metastasis sequencing group.

## Supplementary Materials

The following are available online at https://www.mdpi.com/2072-6694/13/5/1049/s1, Figure S1: Higher magnification photos demonstrating subcellular distribution of PDCD4 by pathology. Figure S2: PDCD4 expression and 5-year survival differences in melanoma based on subcellular localization. Table S1: Genes correlated with PDCD4 Expression in T cells. Table S2: Genes correlated with PDCD4 Expression in B cells.
