# Supplementary material for: Clinical Significance of PDCD4 in Melanoma by Subcellular Expression and in Tumor-Associated Immune Cells
Source: Cancers (Basel). 2021 Mar 2;13(5):1049. doi: 10.3390/cancers13051049 (PMC7958624; doi:10.3390/cancers13051049)
Supplement: Supplementary file 1 [file cancers-13-01049-s001.pdf]

Supplementary Materials: Clinical Significance of PDCD4 in Melanoma by Subcellular Expression and in Tumor-Associated Immune Cells

Thuy T. Tran, Chetan K. Rane, Christopher R. Zito, Sarah A. Weiss, Shlomit Jessel, Liliana Lucca, Benjamin Y. Lu, Victor O. Oria, Adebowale Adeniran, Veronica L. Chiang, Sacit Bulent Omay, David A. Hafler, Harriet M. Kluger and Lucia B. Jilaveanu

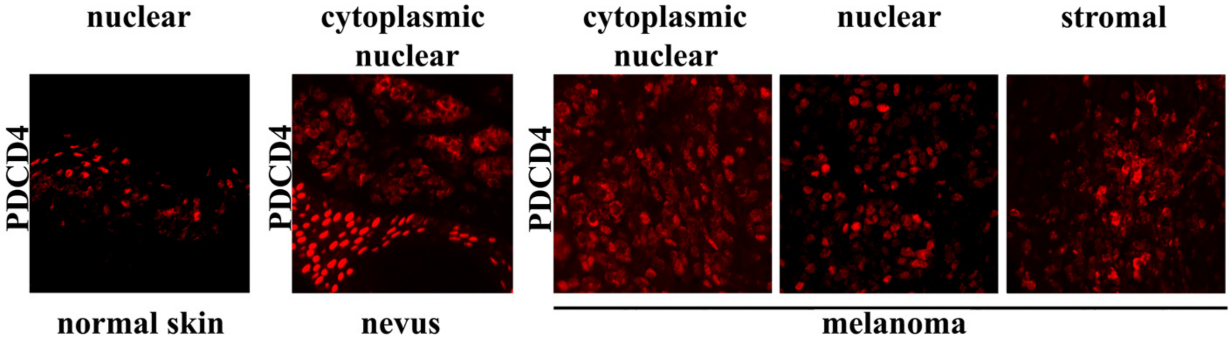

Figure S1. Higher magnification photos demonstrating subcellular distribution of PDCD4 by pathology. PDCD4 is primary located in the nucleus in normal skin but redistributes to become primarily cytoplasmic in nevi. In melanoma, PDCD4 staining can be found as either nuclear, stromal, or a combination of cytoplasmic and nuclear. Magnification 40×.

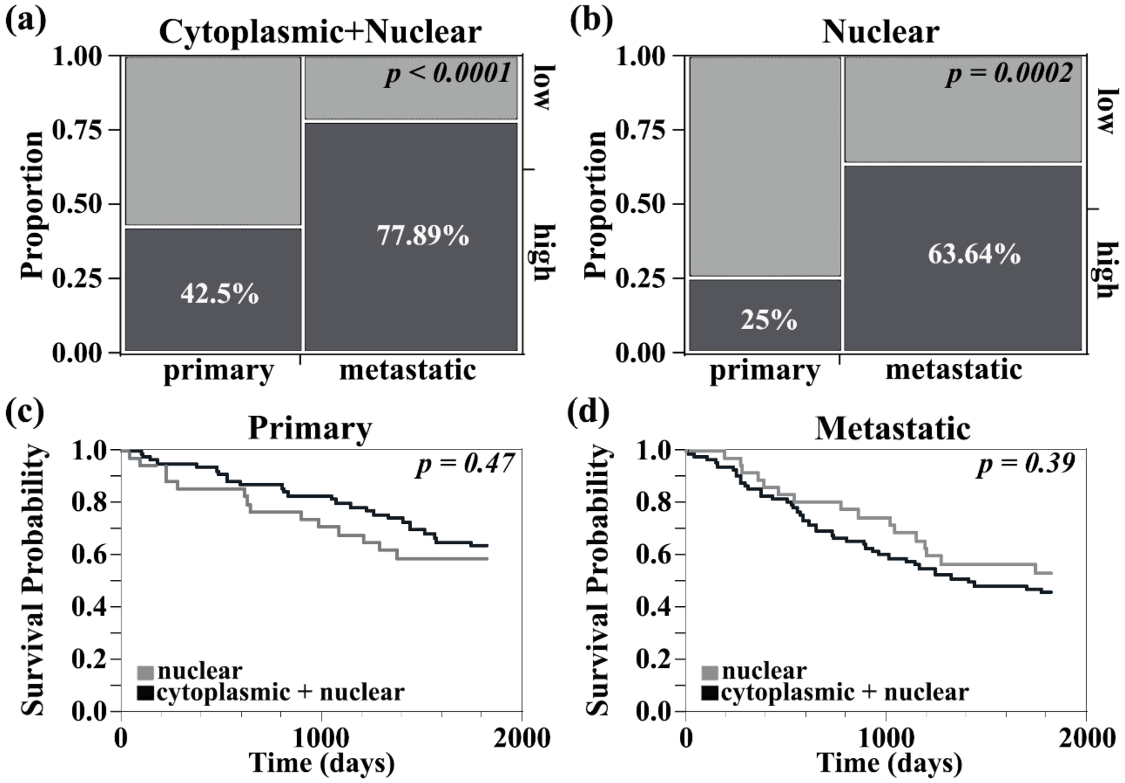

Figure S2. PDCD4 expression and 5-year survival differences in melanoma based on subcellular localization. (**a**,**b**), Metastatic melanoma had more cases with high PDCD4 expression compared to primary melanoma regardless of site of subcellular localization, either cytoplasmic and nuclear (**a**) or predominantly nuclear PDCD4 (**b**). (**c**), No significant 5-year

survival differences were seen in primary melanoma when analyzing PDCD4 expression based on cytoplasmic and nuclear PDCD4 (black line) versus nuclear PDCD4 (gray line) subcellular localization (log-rank $p = 0.47$). (**d**) No significant 5-year survival differences were seen in metastatic melanoma when analyzing PDCD4 based on cytoplasmic and nuclear PDCD4 (black line) versus nuclear PDCD4 (gray line) subcellular localization (log-rank $p = 0.39$).

Table S1. Genes correlated with PDCD4 Expression in T cells.

Number	Gene	Estimate	Adjusted *p* Value
1	PDCD4	1	0
2	GZMK	0.09529773	7.07×10^{-5}
3	HLA-DQA2	0.09330363	0.00014752
4	HLA-DRB5	0.09011205	0.00046375
5	GZMA	0.08974704	0.00052737
6	CCL5	0.08943572	0.00058825
7	NKG7	0.08549676	0.00227116
8	CST7	0.08471141	0.00295259
9	CD74	0.0829165	0.00533206
10	CAP1	0.08245359	0.00619797
11	OVCA2	0.07941275	0.01632863
12	MS4A1	0.0781364	0.02427101
13	CTSW	0.07768833	0.02785448
14	NRBP1	0.07753325	0.02920922
15	IRF2	0.0765719	0.03913
16	CCL4	0.07600486	0.04642124
17	KLRG1	0.07462392	0.07002907
18	TAF7	0.07405565	0.08276958
19	RPL11	−0.0812737	0.00906279

Table S2. Genes correlated with PDCD4 Expression in B cells.

Number	Gene	Estimate	Adjusted *p* Value
1	PDCD4	1	0
2	GZMA	0.11257208	0.01133394
3	VDAC3	0.10471019	0.06354881
4	DUSP2	0.10370821	0.0784945
5	GCSAM	0.10291975	0.09256318
